# The Oncogenic Role of COL23A1 in Clear Cell Renal Cell Carcinoma

**DOI:** 10.1038/s41598-017-10134-2

**Published:** 2017-08-29

**Authors:** Fujiang Xu, Kun Chang, Jian Ma, Yuanyuan Qu, Huyang Xie, Bo Dai, Hualei Gan, Hailiang Zhang, Guohai Shi, Yao Zhu, Yiping Zhu, Yijun Shen, Dingwei Ye

**Affiliations:** 1 Department of Urology, Fudan University Shanghai Cancer Center, Institutes of Biomedical Sciences, Fudan University, Shanghai, 200032 China; 2 Department of Urology, Fudan University Shanghai Cancer Center, Department of Oncology, Shanghai Medical College, Fudan University, Shanghai, 200032 China; 3 Department of Pathology, Fudan University Shanghai Cancer Center, Department of Oncology, Shanghai Medical College, Fudan University, Shanghai, 200032 China; 4 Department of Urology, Fudan University Shanghai Cancer Center, Institutes of Biomedical Sciences, Department of Oncology, Shanghai Medical College, Fudan University, Shanghai, 200032 China

## Abstract

Clear cell renal cell carcinoma (ccRCC) is the most common adult renal neoplasm and its incidence continues to increase. Collagen is the most abundant extracellular matrix protein in stroma, and contributes to the development and progression of ccRCC. We examined the human collagen type XXIII α1 chain (COL23A1) expression in ccRCC and the relationship between COL23A1 and patients’ survival. We found COL23A1 mRNA was elevated in tumor compared with adjacent normal tissues, which was further validated by TCGA cohort. IHC results from 151 ccRCC cases suggested that high COL23A1 expression correlated with larger tumor size (*P* = 0.017) and advanced T stage (*P* = 0.011). The overall survival (OS) was shorter for ccRCC patients with high COL23A1 expression (*P* = 0.002). In multivariate analysis, high COL23A1 expression was an independent prognostic factor of OS (HR: 3.024, *P* = 0.017). Furthermore, COL23A1 knockdown repressed proliferation of ccRCC cell lines by blocking cell cycle progression. Cell adhesion and migration capacity was also downregulated by knockdown of COL23A1. Our data indicate that COL23A1 may be a novel prognostic indicator in ccRCC and might be a specific and accessible biomarker as well as a potential new target for clinical diagnosis of ccRCC.

## Introduction

Renal cell carcinoma (RCC) is the second leading cause of death among all types of urologic cancer and accounts for approximately 3% of all adult malignancies^1,2^. The most common subtype of RCC is clear cell renal cell carcinoma (ccRCC), which accounts for 75–80% of all diagnosed cases^3^. ccRCC is characterized by extremely high rates of local invasion, malignancy, and mortality, and resistance to chemotherapy and radiotherapy^4^. Approximately 25–30% of ccRCC patients present with metastatic disease at the time of diagnosis^5^. Recent advancements in ccRCC treatment strategies, including targeted therapies, have been achieved great therapeutic improvements^6,7^; however, most treated patients eventually develop progressive disease due to acquired resistance^8,9^, resulting in poor prognosis. Insight into the mechanisms of ccRCC pathogenesis and progression may contribute to the development of novel strategies for the treatment of ccRCC.

The extracellular matrix (ECM) is a highly organized structure with many physiological and pathological roles^10^. Modification of the ECM composition through a large array of molecules has been proven to be crucial for tumor initiation and progression^11^. Collagens are the major constituent of the tumor ECM component and play a crucial role in the development of tumors^12–14^. The human collagen type XXIII α1 chain (encoded by *COL23A1*) is a member of the transmembrane collagens, a subfamily of the nonfibrillar collagens^15^. The correlation between upregulation of collagen XXIII and tumor progression has been previously investigated in several types of cancer^15–18^. Wozniak *et al*. also reported that COL23A1 was highly expressed in ccRCC^19^. However, no functional analysis was performed in ccRCC in the previous study. Therefore, the primary aim of this study was to investigate whether COL23A1 expression is correlated with clinicopathological features of ccRCC and patient survival. In addition, we sought to determine the function of COL23A1 in ccRCC using *in vitro* experiments.

## Results

### COL23A1 is strongly upregulated in ccRCC tissues

**Figure 1:**
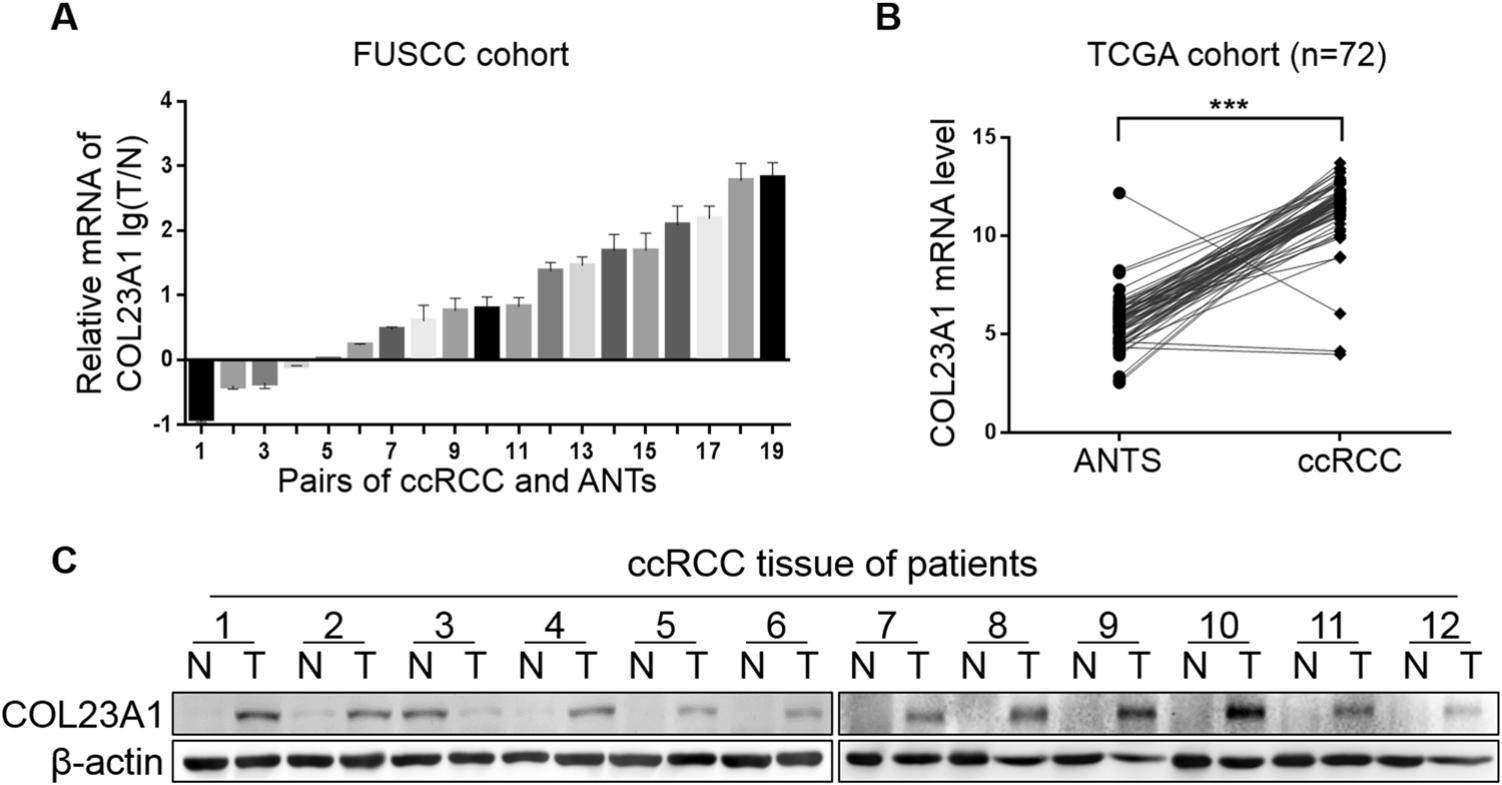
COL23A1 expression in ccRCC tissues and paired ANTs. (**A**) Results of qRT-PCR exhibited the relative mRNA levels of COL23A1 in primary non-metastatic ccRCC tissues compared with paired ANTs from FUSCC cohort. Error bars are ± SD (n = 3). (**B**) The expression of COL23A1 mRNA in primary non-metastatic ccRCC tissues compared with paired ANTs from TCGA cohort. (**C**) Results of western blot analysis showed the COL23A1 protein levels in primary non-metastatic ccRCC tissues compared with paired ANTs from FUSCC cohort.

To investigate the COL23A1 expression alterations in ccRCC, mRNA expression profiling was performed with 19 pairs of primary non-metastatic ccRCC tissue samples and adjacent normal tissues (ANTs). The tissue profiles demonstrated that COL23A1 mRNA expression was upregulated in primary non-metastatic ccRCC compared with paired normal tissues (Fig. 1A). Subsequent validation was conducted by analyzing sequencing results from a published database—the Cancer Genome Atlas (TCGA). As expected, COL23A1 expression was significantly increased in 72 ccRCC tissues than paired ANTs (*P* < 0.001, Fig. 1B).

**Figure 2:**
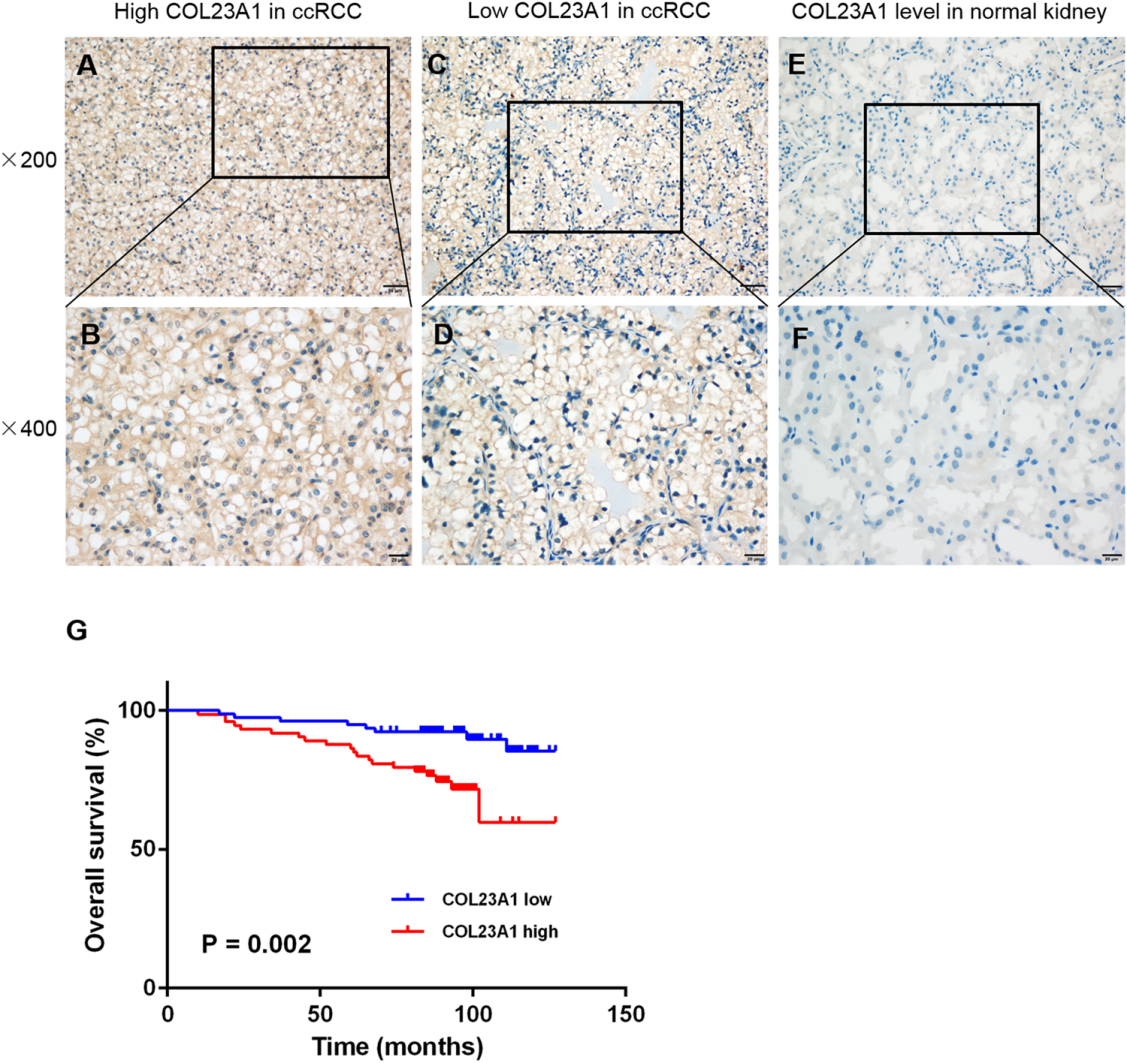
The correlation between COL23A1 expression and prognosis in ccRCC. (**A**,**B**) Representative high COL23A1 level in ccRCC tissues with (**A**) ×200 magnification and (**B**) ×400 magnification. (**C,D**) Representative low COL23A1 level in ccRCC tissues with (**C**) ×200 magnification and (**D**) ×400 magnification. (**E,F**) Representative COL23A1 level in normal kidney tissues with (**E**) ×200 magnification and (**F**) ×400 magnification. (**G**) Kaplan-Meier curves for OS after radical nephrectomy.

Similar to the profiling data, among 12 pairs of comparative freshly collected tissues, 11 paired specimens indicated elevated protein expression levels of COL23A1 in ccRCC tissues compared with corresponding ANTs (Fig. 1C). Furthermore, immunohistochemistry (IHC) performed in 151 ccRCC tissue samples revealed that COL23A1 protein was mainly localized in cytoplasm with or without cytomembrane staining (Fig. 2A–D), while COL23A1 expression was low in normal kidney tissues (Fig. 2E,F).

### Correlation between COL23A1 expression levels and patient characteristics

For the 151 ccRCC tissues evaluated by IHC, COL23A1 expression levels and clinicopathological parameters are listed in Table 1. COL23A1 expression status was not associated with age (*P* = 0.316), sex (*P* = 0.420), Eastern Cooperative Oncology Group (ECOG) performance (*P* = 0.286), International Society of Urological Pathology (ISUP) grade (*P* = 0.877), and necrosis (*P* = 0.914). There was a significant correlation between high COL23A1 expression and larger tumor size (*P* = 0.017). For tumor diameter ≤ 4 cm, only 41.7% (43/103) of tumors had high COL23A1 expression; however, this proportion increased to 62.5% (30/48) for tumors larger than 4 cm (*P* = 0.017). COL23A1 expression was more common in tumors with advanced T stage (70.4%, 19/27) than in T1 stage tumors (43.5%, 54/124; *P* = 0.011).

**Table 1:** Clinicopathological characteristics in relation to COL23A1 expression status.

**Variable**	Entire group (n = 151)	COL23A1 expression		*P* value
		Low expression (n = 65)	High expression (n = 85)	
Age at surgery (year)				0.316
≤50	62	29	33	
>50	89	49	40	
Sex				0.420
Female	51	24	27	
Male	100	54	46	
Tumor size (cm)				**0.017**
≤4	103	60	43	
>4	48	18	30	
ECOG				0.286
0	127	68	59	
1	24	10	14	
T stage				**0.011**
T1	124	70	54	
T2-T4	27	8	19	
ISUP grade				0.877
1	32	16	16	
2	92	49	43	
3–4	27	13	14	
Necrosis				0.914
Absent	141	73	68	
Present	10	5	5	

ISUP, International Society of Urological Pathology.

### Clinical outcome according to COL23A1 expression

Kaplan–Meier survival analysis was applied to compare overall survival (OS) according to COL23A1 expression. Patients with high COL23A1 expression had significantly worse OS (*P* = 0.002; Fig. 2G). The prognostic value of each clinicopathological factor, including COL23A1 expression status, was evaluated for OS (Table 2). Univariate Cox proportion hazard ratio (HR) analysis showed that tumor size (HR: 3.437, *P* = 0.001), ECOG performance (HR: 6.259, *P* = 0.000), T stage (HR: 6.449, *P* = 0.000), ISUP grade (HR: 2.944, *P = *0.001), necrosis (HR: 4.628, *P* = 0.002), and COL23A1 expression status (HR: 3.459, *P* = 0.004) were associated with OS. Further, ECOG performance (HR: 4.762, *P* = 0.000), T stage (HR: 2.724, *P* = 0.037), ISUP grade (HR: 2.077, *P* = 0.024), and COL23A1 expression (HR: 3.024, *P* = 0.017) were identified as independent prognostic factors of OS by multivariate analysis.

**Table 2:** Univariate and multivariate Cox regression analyses of OS in 151 ccRCC patients.

Covariates	Univariate analysis		Multivariate analysis	
	HR (95%CI)	*P* value	HR (95%CI)	*P* value
Age at surgery ( > 50 vs ≤ 50)	1.034(0.484~2.211)	0.930	—	—
Sex (Male vs Female)	1.174(0.530~2.603)	0.692	—	—
Tumor size ( > 4vs ≤ 4)	**3.437(1.623~7.280)**	**0.001**	1.563(0.629~3.879)	0.336
ECOG (1vs 0)	**6.259(2.918~13.426)**	**0.000**	**4.762(2.093~10.836)**	**0.000**
T stage (T2-4 vs T1)	**6.449(3.034~13.707)**	**0.000**	**2.724(1.016~6.995)**	**0.037**
ISUP grade (2 or 3-4 vs 1)	**2.944(1.561~5.552)**	**0.001**	**2.077(1.103~3.910)**	**0.024**
Necrosis (Present vs Absent)	**4.628(1.747~12.262)**	**0.002**	2.277(0.738~7.019)	0.152
COL23A1 expression (High vs Low)	**3.459(1.494~8.012)**	**0.004**	**3.024(1.221~7.489)**	**0.017**

ISUP, International Society of Urological Pathology.

### Downregulation of COL23A1 by small interfering RNA (siRNA) in ccRCC cell lines

**Figure 3:**
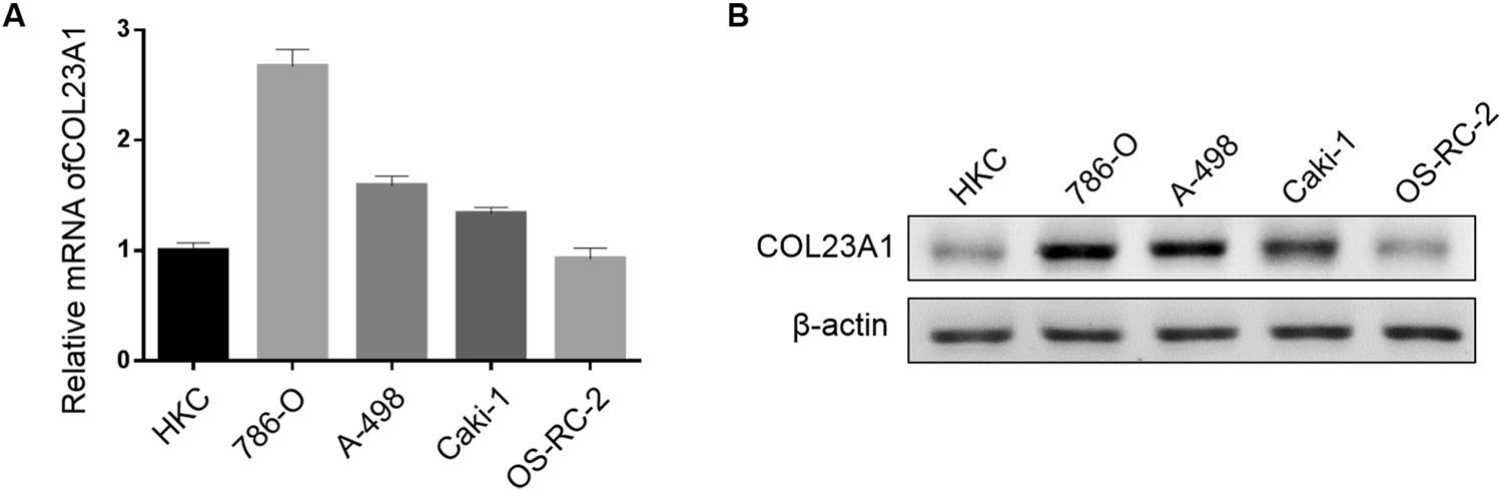
The mRNA and protein expression of COL23A1 in normal and ccRCC cell lines. (**A**) A statistical analytical graph showed COL23A1 mRNA levels in the human kidney cortex cell lines (HKC) and four ccRCC cell lines (786-O, A-498, Caki-1, OS-RC-2). Error bars are ± SD (n = 3). (**B**) Results of western blot analysis showed COL23A1 protein levels in the human kidney cortex cell lines (HKC) and four ccRCC cell lines (786-O, A-498, Caki-1, OS-RC-2).

**Figure 4:**
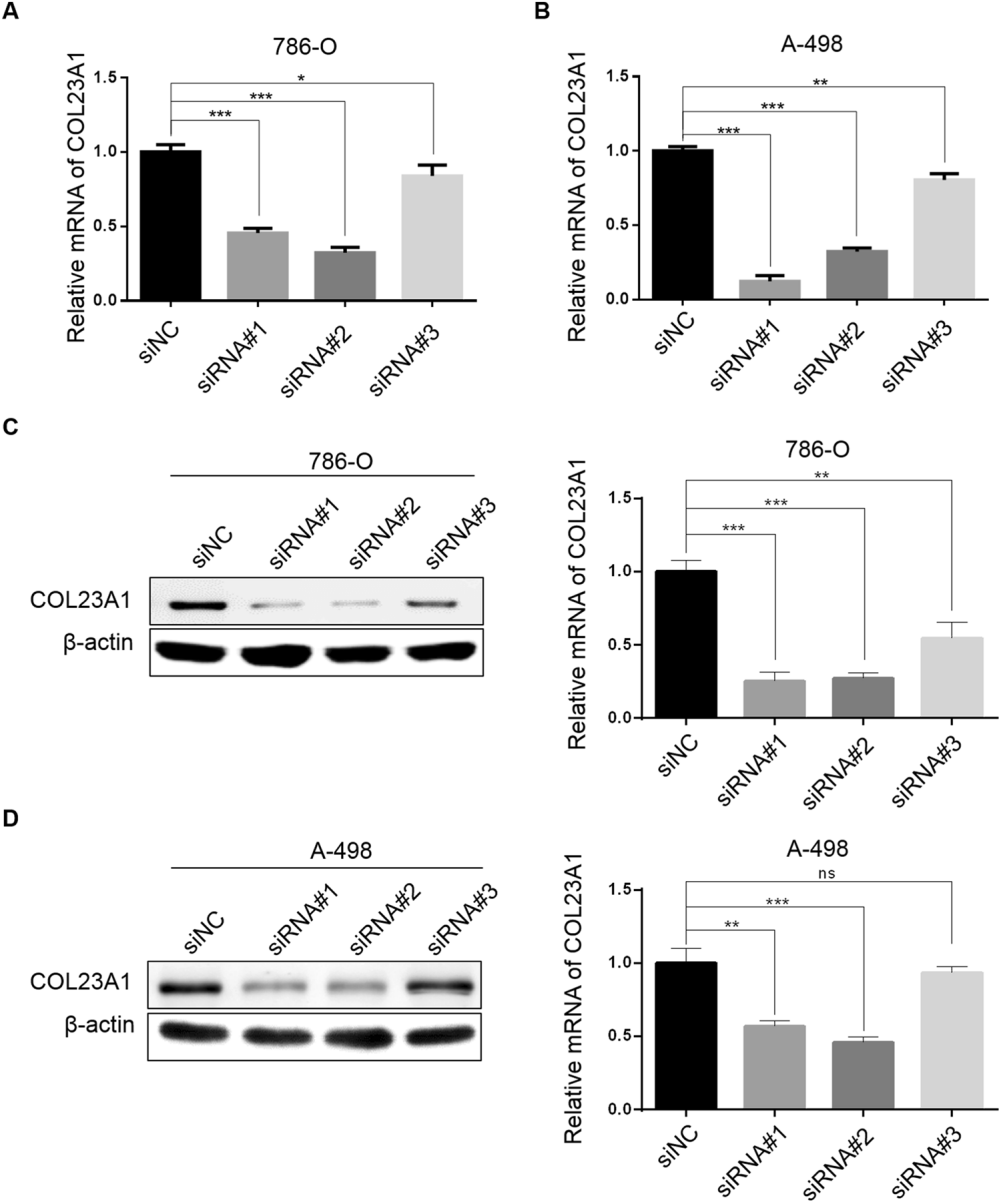
COL23A1 was down-regulated by small interfering RNA (siRNA) in ccRCC cell lines. (**A**,**B**) Relative COL23A1 mRNA levels in **(A)** 786-O and (**B**) A-498 cells that were transfected with siRNAs (#1, #2, #3) compared with siNC respectively were analyzed using qRT-qPCR. Error bars are ± SD (n = 3). (**C**) Results of western blot analysis showed the COL23A1 protein level of 786-O cells that were transfected with siRNAs (#1, #2, #3) compared with siNC, and the histogram right was quantification of the western blot analysis results. Error bars are ± SD (n = 3). (**D**) Results of western blot analysis showed the COL23A1 protein level of A-498 cells that were transfected with siRNAs (#1, #2, #3) compared with siNC, and the histogram right was quantification of the western blot analysis results. Error bars are ± SD (n = 3).

To confirm the above data derived from human ccRCC tissues and to corroborate the function of COL23A1 in ccRCC cells, the COL23A1 expression level was detected in a panel of ccRCC cell lines as well as the human renal proximal tubular epithelial cell line (HKC). As shown in Fig. 3A, COL23A1 mRNA was expressed at a relatively high level in 786-O and A-498 cells. Similarly, a comparatively high level of COL23A1 protein expression was observed in 786-O and A-498 cell lines (Fig. 3B). Decreased levels of COL23A1 mRNA (Fig. 4A,B) and protein (Fig. 4C,D) were observed in 786-O and A-498 cell lines after treatment with three siRNA sequences specifically targeting COL23A1 mRNA. As shown in Fig. 4, siRNA#1 and siRNA#2 demonstrated the highest interference efficiency, and were therefore selected for use in further experiments.

### Roles of COL23A1 in ccRCC cell lines

**Figure 5:**
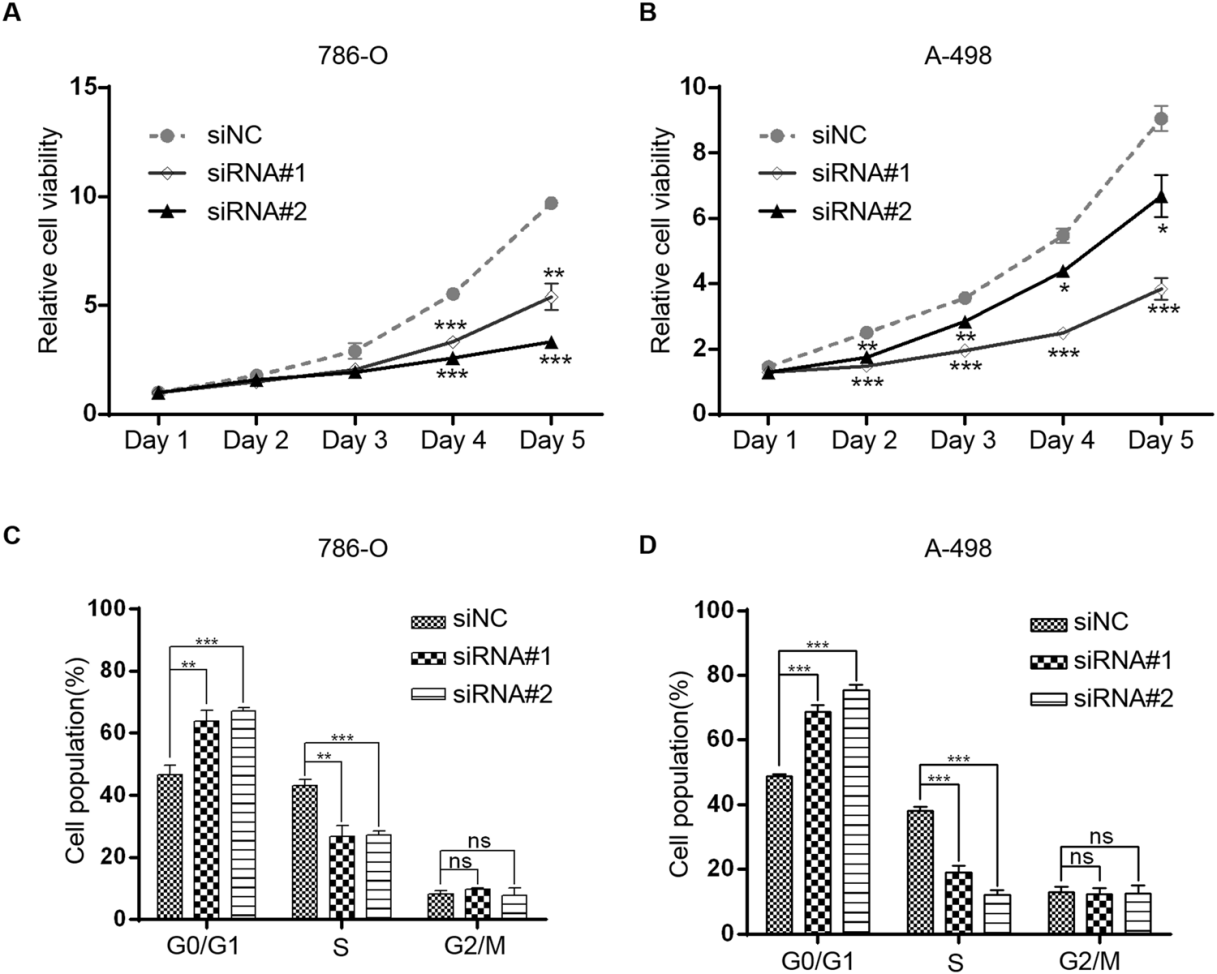
Effect of COL23A1 silencing on cell proliferation and cell cycle in ccRCC cells. (**A**) CCK-8 assays showed the effect of COL23A1 knockdown on the proliferation of 786-O cells. Error bars are ± SD (n = 3). (**B**) CCK-8 assays showed the effect of COL23A1 knockdown on the proliferation of A-498 cells. Error bars are ± SD (n = 3). (**C**) Effect of COL23A1 silencing on the cell cycle of 786-O cells. Error bars are ± SD (n = 3). (**D**) Effect of COL23A1 silencing on the cell cycle of A-498 cells. Error bars are ± SD (n = 3).

**Figure 6:**
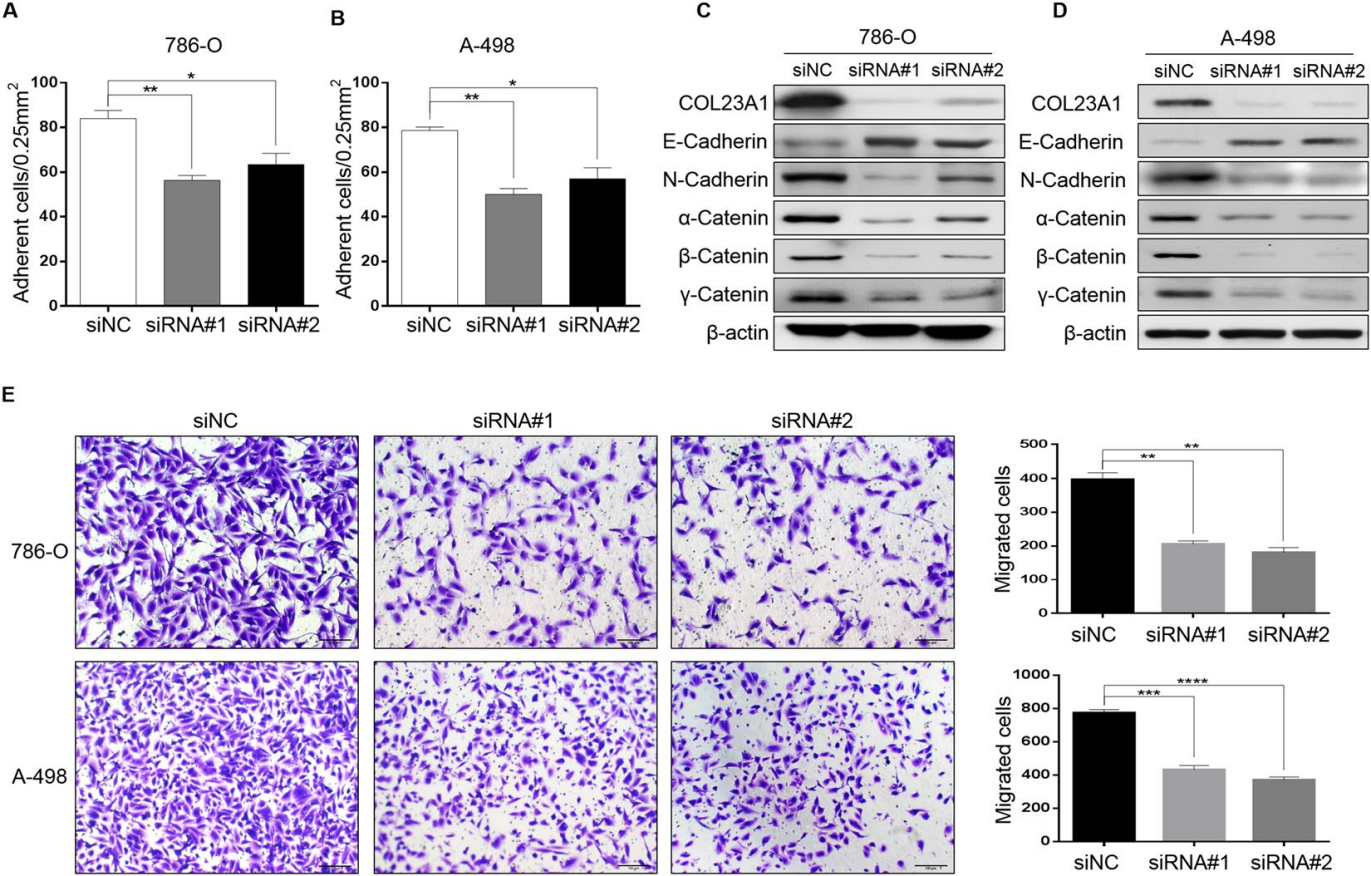
Effect of COL23A1 silencing on cell migration and adhesion in ccRCC cells. **(A, B)** Effect of COL23A1 silencing on the cell adhesion of **(A)** 786-O and **(B)**A-498 cells. Error bars are ± SD (n = 3). **(C, D)** Effect of COL23A1 silencing on the expression of proteins involved in cell adhesion of **(C)** 786-O and **(D)** A-498. **(E)** Representative images of migration assays performed using 786-O (upper left) and A-498 (lower left) cells (scale bars, 100 µm). The histograms right were quantification of the migration assays of 786-O (upper) and A-498 (lower) cells. Error bars are ± SD (n = 3).

The CCK-8 assay was conducted to assess ccRCC cell proliferation *in vitro*. Proliferation of 786-O cells treated with siRNA#2 (Fig. 5A) and of A-498 cells treated with siRNA#1 (Fig. 5B) showed obvious suppression of proliferation compared with cells treated with siNC. Next, a fluorescence-activated cell sorting assay was employed to investigate whether the inhibition of ccRCC cell proliferation upon knockdown of COL23A1 is associated with cell cycle arrest. Flow cytometry results indicated that COL23A1 knockdown ccRCC cells had a larger G0/G1 population and a decreased S phase population compared with the siNC group for both cell lines (Fig. 5C), indicating that COL23A1 knockdown blocked cell cycle progression. To examine whether COL23A1 is involved in cell adhesion, we measured ccRCC cells adhesion to fibronectin, one of extracellular matrix components. Knockdown of COL23A1 in 786-O cells resulted in decreasing adhesive ability (Fig. 6A). Similar results were obtained in A-498 cells (Fig. 6B). Therefore, western blot analysis was used to examine changes in a series of proteins with known roles in cell adhesion, including E-cadherin, N-cadherin, α-catenin, β-catenin and γ-catenin^20–22^. Silencing of COL23A1 in 786-O (Fig. 6C) and A-498 (Fig. 6D) cells led to decreased protein levels of N-cadherin, α-catenin, β-catenin and γ-catenin, while increased protein level of E-cadherin. These results revealed a pivotal role of COL23A1 in cell adhesion in ccRCC. Furthermore, Transwell assays were also performed to measure the migration ability of ccRCC cells. COL23A1 RNA interference of 786-O and A-498 cells remarkably attenuated the migration ability by 50–55% compared with the corresponding control (Fig. 6E).

## Discussion

In the present study we investigated the expression pattern, clinical significance, and biological functions of COL23A1 in ccRCC, including proliferation, cell cycle progression, cell adhesion and migration. COL23A1 expression was elevated in ccRCC tissues compared with ANTs and was associated with poor prognosis of ccRCC patients. Moreover, we demonstrated that knockdown of COL23A1 inhibited ccRCC cell proliferation, which was correlated with inhibition of cell cycle progression, and also attenuated the cell adhesion and migration capability of ccRCC cells.

Investigations of COL23A1 in many malignancies have implicated an important role in tumor maintenance^15–18^. Banyard *et al*. originally discovered that collagen XXIII is upregulated in the highly metastatic rat prostate carcinoma subline AT6.1 compared with lower metastatic potential sublines^15^, suggesting a close correlation between collagen XXIII expression and prostate cancer metastatic behavior. Furthermore, collagen XXIII was experimentally validated as a reliable biomarker for recurrence and distant metastases in prostate cancer patient samples. Moreover, a reduced level of collagen XXIII in urine of prostate cancer patients was reported after prostatectomy^16^. The same study also reported that COL23A1 was upregulated in prostate cancer tissues, and might be an independent predictor of PSA-defined disease recurrence^16^. Spivey *et al*. showed that COL23A1 had potential as a tissue and urinary biomarker for non-small cell lung cancer^17^.

Several studies have also showed clues about the relationship between COL23A1 expression and ccRCC. One previous research stated that COL23A1 mRNA distribution was restricted and expression was very low in mouse tissues, and signals were very low in human kidney^23^. Another study reported that collagen XXIII protein could be detected in samples of ccRCC patients using immunohistochemical staining^17^. By conducting whole-genome expression profiling on 101 pairs of ccRCC tumors and adjacent non-tumor renal tissues, Wozniak *et al*. identified 1650 significantly differential expression genes. When matching them with the TCGA RNA-Seq dataset, they found that COL23A1 were overexpressed in both datasets^19^. We observed a higher level of COL23A1 mRNA and protein in clinical ccRCC tissues compared with matched ANTs. The mRNA level of COL23A1 was further validated by TCGA database, which showed significantly increased COL23A1 expression in 72 ccRCC tumor tissues than paired ANTs. In addition to validation by western blot analysis at the protein level, IHC results demonstrated that high protein expression of COL23A1 was significantly correlated with poor prognosis of patients with ccRCC (*P* = 0.002). The above results demonstrated that COL23A1 might function as an oncogene, playing an important role in the tumorigenesis and progression of ccRCC.

Most studies on COL23A1 revealed a close relationship with cell adhesion, invasion, and metastasis. Exogenously expressed recombinant COL23A1 competed with corneal epithelial cell surface COL23A1 for binding to collagen IV and Matrigel^24^, and COL23A1 directly interacted with integrin α2β1 and sufficiently induced integrin α2β1-dependent attachment and spreading of keratinocytes^25^, corroborating the results of Spivey *et al*., who reported that COL23A1 played a role in cancer cell adhesion, anchorage-independent growth, and metastasis^17^. Our research demonstrated that knockdown of COL23A1 attenuated ccRCC cell adhesion and migration *in vitro*. Moreover, COL23A1 promoted ccRCC cell proliferation and cell cycle progression. Three days after silencing of COL23A1, the proliferation of 786-O and A-498 cells was significantly inhibited compared with cells in control groups. Furthermore, knockdown of COL23A1 induced cell cycle arrest in G0/G1 phase, which may partially account for the inhibitory effect on the proliferation of ccRCC cells.

To the best of our knowledge, this is the first comprehensive study establishing that COL23A1 plays an oncogenic role in ccRCC tumorigenesis and progression and has the potential to be a novel biomarker for predicting prognosis of ccRCC patients. However, some limitations of our study should be addressed. First, this was a single-center pilot study and the prognostic value of COL23A1 was not validated in another independent cohort. Second, the exact mechanism by which COL23A1 promotes ccRCC progression was not investigated. Thus, large-scale examinations of COL23A1 expression in ccRCC patients and further explorations to elucidate the underlying molecular mechanisms are required.

In conclusion, our results demonstrated that COL23A1 expression is significantly increased in ccRCC tissues. COL23A1 overexpression was correlated with more aggressive tumor behavior. In addition, upregulation of COL23A1 expression was associated with poor prognosis of ccRCC. *In vitro* experiments showed that COL23A1 knockdown repressed proliferation by blocking cell cycle progression, and also attenuated the cell adhesion and migration capacity. These findings suggest that COL23A1 may be a novel prognostic indicator in ccRCC and might also be a specific and accessible biomarker as well as a potential new target for clinical diagnosis of ccRCC.

## Materials and Methods

### Patients and tissue samples

Tissue specimens were obtained from patients with ccRCC who underwent partial or radical nephrectomy from January 2005 to December 2008 at Fudan University Shanghai Cancer Center (FUSCC). None of the patients received preoperative or postoperative adjuvant anticancer therapy. A total of 151 patients with localized ccRCC were included in the IHC analysis. All patients had paraffin-embedded tissue blocks available for IHC staining and outcome data. We also included 31 pairs of comparative freshly collected tissues from localized ccRCC, among which 19 were analyzed by mRNA expression profiling and 12 were subjected to protein expression analysis by western blot. Patient medical records were reviewed to collect demographic data, ECOG performance status, pathological findings, clinical outcomes, and follow-up information. Tumor sizes were defined as the largest diameter of the surgically removed tumor mass. Patients received regular follow-up through telephone calls or clinic visits every 3 months until August 2015 or until the date of death. The study was carried out in accordance with the ethical standards of the Declaration of Helsinki II and approved by the Institution Review Board of Fudan University Shanghai Cancer Center. Informed consent was obtained from each patient and the study was approved by our Institutional Ethics Committee. A data set from an independent cohort in the TCGA database was used for the validation of COL23A1 expression.

### Cell lines and cell culture

Four human ccRCC cell lines (786-O, A-498, Caki-1, OS-RC-2) and HKC were preserved in our laboratory. According to the American Type Culture Collection, 786-O and OS-RC-2 were cultured in RPMI-1640 medium (HyClone, Logan, UT, USA), A-498 were cultured in Dulbecco’s modified Eagle’s medium (HyClone, Logan, UT, USA), and Caki-1 was cultured in McCoy’s 5 A (Modified) Medium (Gibco, Carlsbad, CA, USA). All media were supplemented with 10% fetal bovine serum (FBS, Gibco, Carlsbad, CA, USA) and 1% penicillin–streptomycin solution. Cells were maintained in 5% CO_2_ at 37 °C.

### siRNA transfection

Three siRNAs specifically targeting COL23A1 mRNA sequences (#1: 5′-GGACUAGCGAAGAUCCGGA-3′, #2: 5-CGAGGGUCUAGCUCUACCA-3′, and #3: 5′-GGAAGCUCCAUCCGAAUGU-3′) and mock siRNA negative control (siNC: 5′-UUCUCCGAACGUGUCACGU-3ʹ) were chemically synthesized (Biotend, Shanghai, China). For transfection, ccRCC cells were seeded in six-well plates at 50–70% confluency. COL23A1 siRNAs were transfected with Lipofectamine 2000 (Invitrogen, Carlsbad, CA, USA) according to the manufacturer’s protocol. At 48 hours after transfection, the cells were used for the following assays.

### RNA extraction and quantitative real-time polymerase chain reaction

A total of 19 pairs of ccRCC and ANTs were randomly collected during surgery of ccRCC patients. Total RNA was extracted from the cell lines and patient samples using TRIzol Reagent (Invitrogen, Carlsbad, CA, USA) according to the manufacturer’s protocol. After quantification using NanoDrop-2000 (Thermo Fisher Scientific, Wilmington, DE, USA), total RNA was reverse transcribed to cDNA using PrimeScript RT Master Mix (Takara, Shiga, Japan) and subjected to qRT-PCR using an ABI Prism 7900 HT Sequence Detection System (Applied Biosystems, Foster City, CA, USA). β-actin was used as the endogenous control to calculate relative gene expression levels by the comparative cycle threshold (CT) (2^-ΔΔCT^) method. The following primers were used in this study: 5′-AGGTTCTTACAGGGCAGTA-3′ (forward) and 5′-TAACATTCAAGAGTATGCCCACC-3′ (reverse) for COL23A1; 5′-CTACGTCGCCCTGGACTTCGAGC-3′ (forward) and 5′-GATGGAGCCGCCGATCCACACGG-3ʹ (reverse) for β-actin.

### Western blot analysis

Standard procedures were followed for western blot analysis. The primary antibodies used for western blotting were anti-COL23A1 (1:800, 14337–1-AP, ProteinTech Group, Inc., Chicago, IL), anti-E-cadherin (1: 1,000, #14472, Cell Signaling Technology, Danvers, MA, USA), anti-N-cadherin (1: 1,000, #4061, Cell Signaling Technology, Danvers, MA, USA), anti-α-catenin (1: 1,000, #2131, Cell Signaling Technology, Danvers, MA, USA), anti-β-catenin (1: 1,000, #9562, Cell Signaling Technology, Danvers, MA, USA), anti-γ-catenin (1: 1,000, #2309, Cell Signaling Technology, Danvers, MA, USA) and anti-β-actin (1: 5,000, AC004, ABclonal, Cambridge, MA, USA) antibodies.

### Immunohistochemistry analysis

Tissue sections were deparaffinized in xylene and rehydrated in graded alcohols. Endogenous peroxidase was blocked by incubation with 3% hydrogen peroxide (Sigma-Aldrich, USA) diluted in methanol for 15 min at 15 °C. Heat-induced antigen retrieval was conducted in 10 mM citrate buffer solution (pH = 6.0). Immunohistochemical staining was performed using an avidin-biotin-peroxidase complex method. Slides were blocked using normal goat serum followed by the avidin/biotin blocking kit and incubated with anti-COL23A1 antibody (1:200, Catalog # MAB4165, R&D Systems, Minneapolis, Minnesota, USA) at 4 °C overnight. Slides were then sequentially incubated with biotinylated goat anti-rabbit IgG for 1 h at 37 °C and with preformed avidin-biotin complex. Specifically bound COL23A1 proteins were visualized by staining with 3,3ʹ-diaminobenzidine tetrahydrochloride. Slides were counterstained with Mayer’s hematoxylin, dehydrated, and mounted.

All slides were examined and scored in an open discussion by two experienced pathologists, who were blinded to the outcome and clinical data. The COL23A1 immunostaining was measured based on the intensity of staining (intensity score) and the quantity of immunoreactive cells (quantity score), as previously reported^26^. The intensity of immune staining was scored as 0, negative; 1, weak; 2, moderate; and 3, intense. The quantity of immunoreactive cells was scored as 0%, none; 1, 1%–30%; 2, 31%–60%; 3, >60%^27^. The product of intensity score and quantity score was used as the total score, in which 0–3 indicates low expression and 4–9 indicates high expression^28^.

### Cell proliferation assay

786-O and A-498 cells were transfected with one of three COL23A1 siRNAs or siNC. A total of 2 × 10^3^ cells were seeded in each well of 96-well plates. After 12 h proliferation was measured using the Cell Counting Kit-8 (Dojindo, Kumamoto, Japan) according to the manufacturer’s instructions. In brief, 10 μL CCK-8 solution was added to each well and the absorbance of each well was measured at 450 nm. All experiments were conducted in triplicate. Cell proliferation curves were plotted using the percentage of absorbance in each treatment group relative to the untreated group at each given time point.

### Flow cytometry analysis of the cell cycle

For cell cycle analysis, cells were harvested and fixed with −20 °C pre-cooled 70% ethanol overnight at 4 °C, followed by three washes in cold phosphate-buffered saline (PBS). Cells were stained with the cell cycle staining buffer (MultiSciences, Hangzhou, China) at room temperature for 30 minutes and analyzed with a Beckman Coulter flow cytometer (Beckman Coulter, Brea, CA, USA).

### Cell Adhesion assay

Six-well plates were coated with human fibronectin protein (50 μg/ml in PBS, Sigma) overnight. Plastic dishes served as the background control. Plates were washed with 1% bull serum albumin in PBS to block nonspecific cell adhesion. Tumor cells (0.5 × 10^6^) were then added to each well for 30 minutes. Subsequently, nonadherent tumor cells were washed off by PBS, and the remaining adherent cells were fixed with 4% paraformaldehyde solution and counted under a microscope. The mean adherent cells were calculated from five different observation fields (5 × 0.25 mm^2^) after adherent cells (coated well) minus adherent cells (background). Each experiment was repeated three times.

### Migration assay

Boyden Transwell chambers (8 μm, 24-well format; Corning Co., New York, NY, USA) containing 8-μm membrane filters were applied to 24-well plates. A total of 2 × 10^4^ cells suspended in 200 µL FBS-free medium were placed in the upper well, and culture medium supplemented with 10% FBS was added to the bottom chamber. After incubation for 24 h or 48 h at 37 °C, the cells on the upper surface were wiped off and cells on the lower surface were fixed with 4% paraformaldehyde solution and stained with 0.1% crystal violet. The average number of migrated cells was counted in five randomly selected fields under a microscope. Experiments were independently repeated in triplicate.

### Statistical analysis

The significance of between-group differences was estimated using Student’s t-test, chi-square test, or Wilcoxon test, as appropriate. Univariate and multivariate Cox regression models were performed to evaluate the prognostic value of all parameters. OS for patient groups classified according to tumor COL23A1 expression level was calculated by the Kaplan–Meier method and compared using the log-rank test. All statistical tests were performed using SPSS version 20 (SPSS Inc., Chicago, IL, USA). Two-tailed *P* values < 0.05 were considered statistically significant. Significance was indicated as *P < 0.05, **P < 0.01, and ***P < 0.001.

## Electronic supplementary material

Supplementary Information
