# Author Correction: The Oncogenic Role of COL23A1 in Clear Cell Renal Cell Carcinoma

**DOI:** 10.1038/s41598-018-23756-x

**Published:** 2018-04-12

**Authors:** Fujiang Xu, Kun Chang, Jian Ma, Yuanyuan Qu, Huyang Xie, Bo Dai, Hualei Gan, Hailiang Zhang, Guohai Shi, Yao Zhu, Yiping Zhu, Yijun Shen, Dingwei Ye

**Affiliations:** 1 Department of Urology, Fudan University Shanghai Cancer Center, Institutes of Biomedical Sciences, Fudan University, Shanghai, 200032 China; 2 Department of Urology, Fudan University Shanghai Cancer Center, Department of Oncology, Shanghai Medical College, Fudan University, Shanghai, 200032 China; 3 Department of Pathology, Fudan University Shanghai Cancer Center, Department of Oncology, Shanghai Medical College, Fudan University, Shanghai, 200032 China; 4 Department of Urology, Fudan University Shanghai Cancer Center, Institutes of Biomedical Sciences, Department of Oncology, Shanghai Medical College, Fudan University, Shanghai, 200032 China

Correction to: *Scientific Reports* 10.1038/s41598-017-10134-2, published online 29 August 2017

In the original version of this Article there were errors with the affiliations which were incorrectly listed as ‘Department of Urology, Fudan University Shanghai Cancer Center, Shanghai, 200032, China’, ‘Institutes of Biomedical Sciences, Fudan University, Shanghai, 200032, China’, ‘Department of Oncology, Shanghai Medical College, Fudan University, Shanghai, 200032, China’ and ‘Department of Pathology, Fudan University Shanghai Cancer Center, Shanghai, 200032, China’.

The correct affiliations are listed below.

Affiliation 1:

Department of Urology, Fudan University Shanghai Cancer Center, Institutes of Biomedical Sciences, Fudan University, Shanghai, 200032, China.

Affiliation 2:

Department of Urology, Fudan University Shanghai Cancer Center, Department of Oncology, Shanghai Medical College, Fudan University, Shanghai, 200032, China.

Affiliation 3:

Department of Pathology, Fudan University Shanghai Cancer Center, Department of Oncology, Shanghai Medical College, Fudan University, Shanghai, 200032, China.

Affiliation 4:

Department of Urology, Fudan University Shanghai Cancer Center, Institutes of Biomedical Sciences, Department of Oncology, Shanghai Medical College, Fudan University, Shanghai, 200032, China.

These errors have now been corrected in the PDF and HTML versions of the Article, and in the accompanying Supplementary Information file.

