# Supplementary material for: The Oncogenic Role of COL23A1 in Clear Cell Renal Cell Carcinoma
Source: Sci Rep. 2017 Aug 29;7:9846. doi: 10.1038/s41598-017-10134-2 (PMC5575106; doi:10.1038/s41598-017-10134-2)
Supplement: Supplementary file 1 — Supplementary Information [file 41598_2017_10134_MOESM1_ESM.pdf]

Title：The Oncogenic Role of COL23A1 in Clear Cell Renal Cell Carcinoma

Fujiang Xu [1*], Kun Chang[2*], Jian Ma[2], Yuanyuan Qu[2], Huyang Xie[2], Bo Dai[2], Hualei Gan[3], Hailiang Zhang[2], Guohai Shi[2], Yao Zhu[2], Yiping Zhu[2] Yijun Shen[2], Dingwei Ye[4]

[1] Department of Urology, Fudan University Shanghai Cancer Center, Institutes of Biomedical Sciences, Fudan University, Shanghai, 200032, China.

[2] Department of Urology, Fudan University Shanghai Cancer Center, Department of Oncology, Shanghai Medical College, Fudan University, Shanghai, 200032, China.

[3] Department of Pathology, Fudan University Shanghai Cancer Center, Department of Oncology, Shanghai Medical College, Fudan University, Shanghai, 200032, China.

[4] Department of Urology, Fudan University Shanghai Cancer Center, Institutes of Biomedical Sciences, Department of Oncology, Shanghai Medical College, Fudan University, Shanghai, 200032, China.

[*] These authors contributed equally to this work.

Correspondence and requests for materials should be addressed to D.Y. (email: dwyeli@163.com)

Supplementary materials for Fig. 1C:

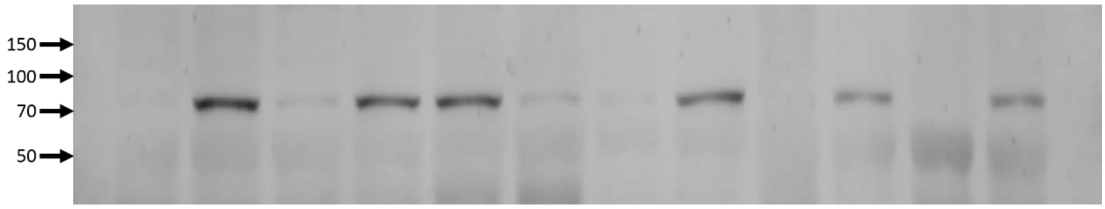

Detecting COL23A1 of ccRCC patients' (1~6) tissues by Western Blot

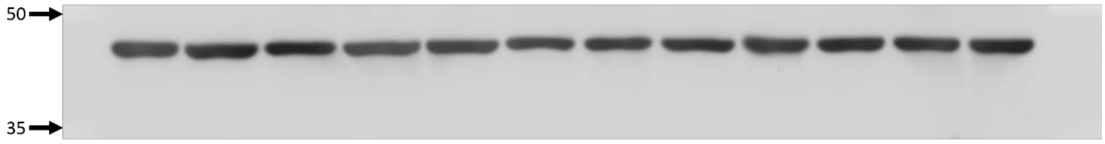

Detecting endogenous control (β-actin) of ccRCC patients' (1~6) tissues by Western Blot

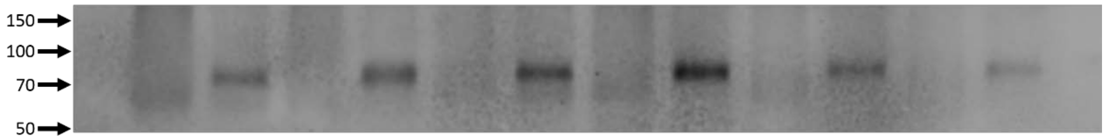

Detecting COL23A1 of ccRCC patients' (7~12) tissues by Western Blot

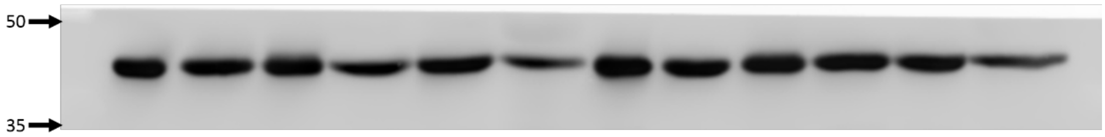

Detecting endogenous control (β-actin) of ccRCC patients' (7~12) tissues by Western Blot

Supplementary materials for Fig. 3B:

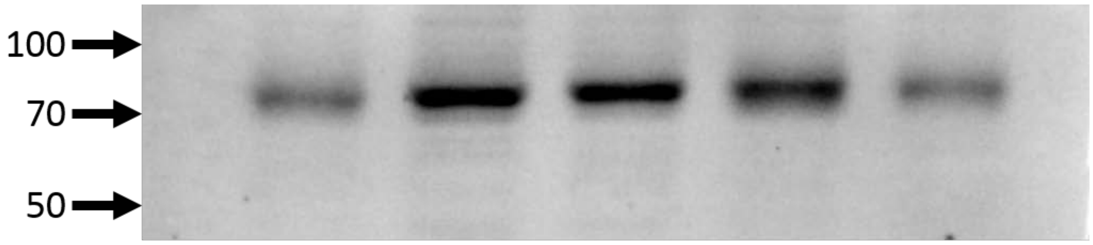

COL23A1 expression in one normal and four ccRCC cell lines

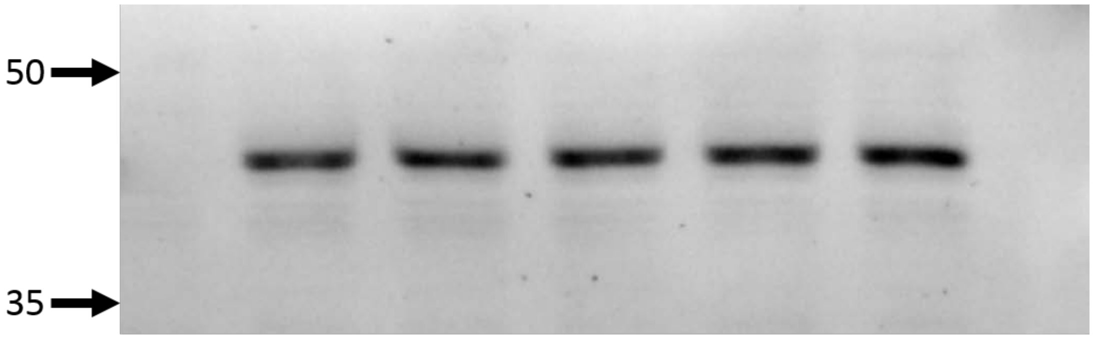

Expression of endogenous control (β-actin) in one normal and four ccRCC cell lines

Supplementary materials for Fig. 4C:

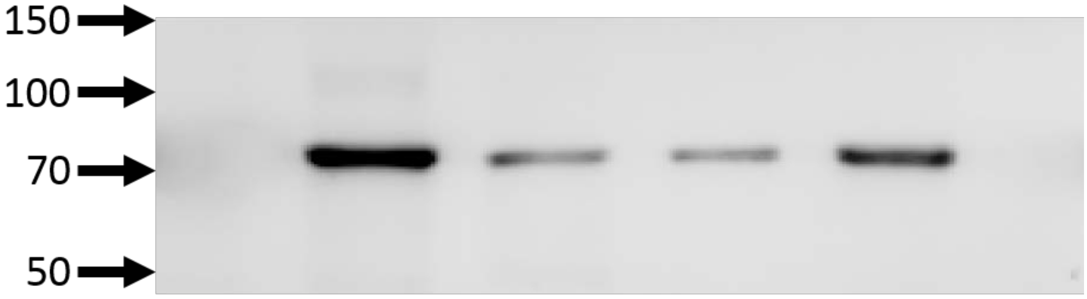

Detecting the efficiency of COL23A1 knockdown by small interfering RNA in 786-O

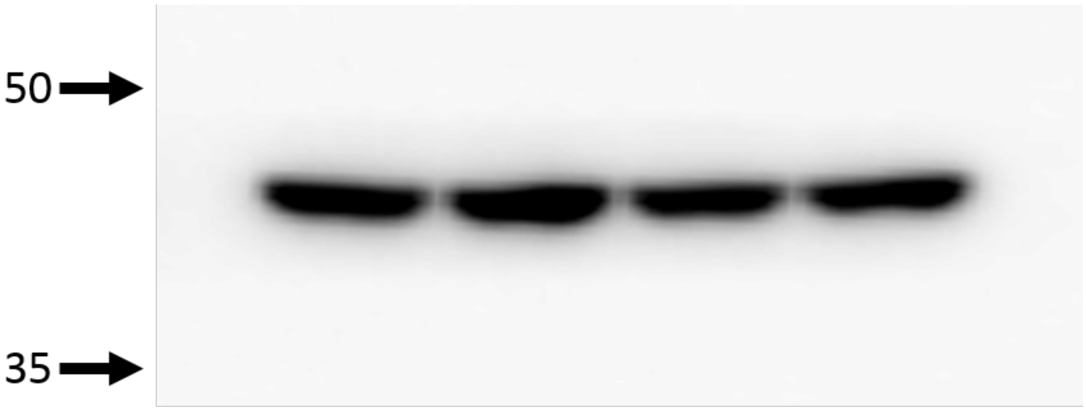

Expression of endogenous control (β-actin) in cell lines detecting the efficiency of COL23A1 knockdown in 786-O

Supplementary materials for Fig. 4D:

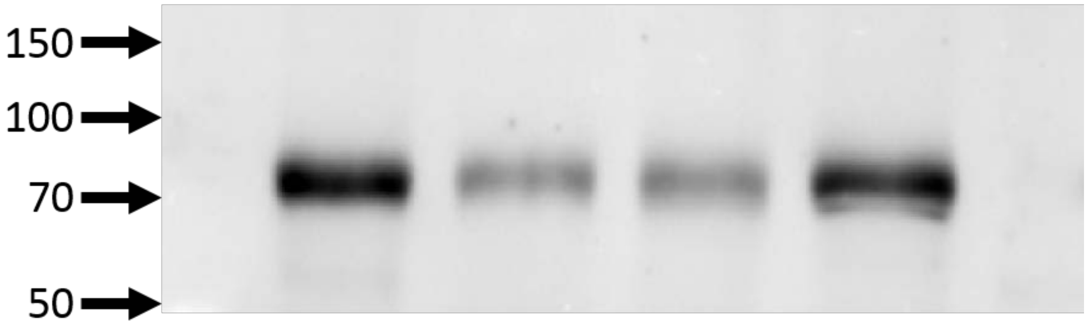

Detecting the efficiency of COL23A1 knockdown by small interfering RNA in A-498

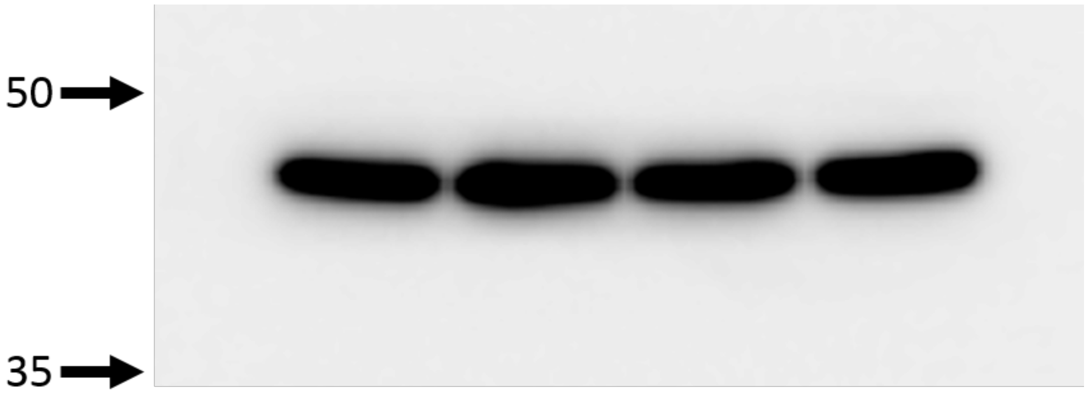

Expression of endogenous control (β-actin) in cell lines detecting the efficiency of COL23A1 knockdown in A-498

Supplementary materials for Fig. 6C:

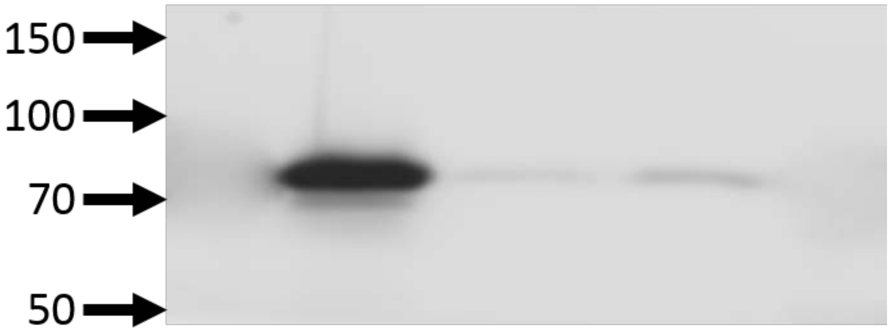

COL23A1 expression in 786-O (siNC, siRNA#1 and siRNA#2)

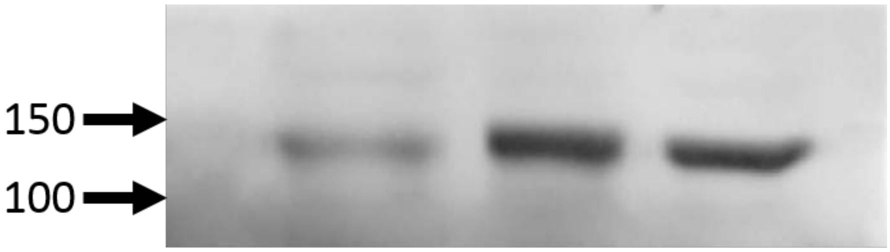

E-cadherin expression in 786-O (siNC, siRNA#1 and siRNA#2)

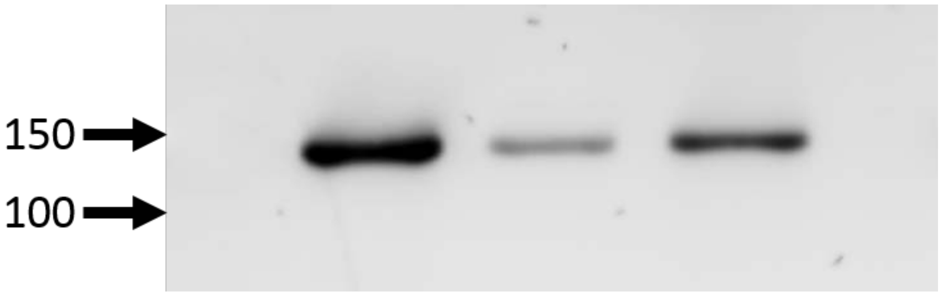

N-cadherin expression in 786-O (siNC, siRNA#1 and siRNA#2)

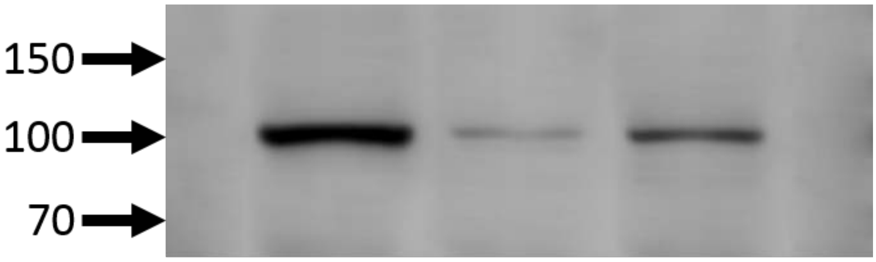

α -catenin expression in 786-O (siNC, siRNA#1 and siRNA#2)

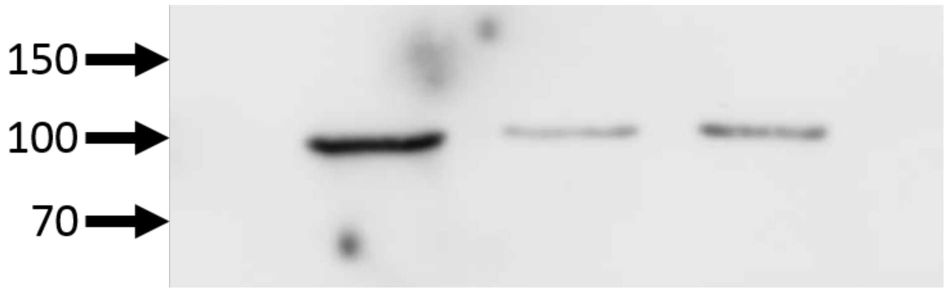

β -catenin expression in 786-O (siNC, siRNA#1 and siRNA#2)

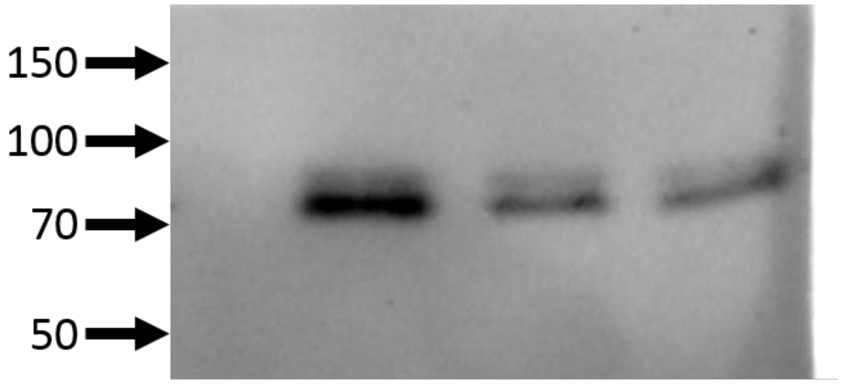

γ -catenin expression in 786-O (siNC, siRNA#1 and siRNA#2)

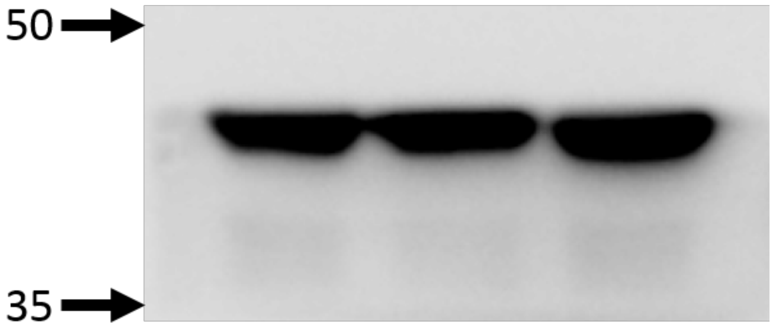

Expression of endogenous control (β-actin) in 786-O (siNC, siRNA#1 and siRNA#2)

Supplementary materials for Fig. 6D:

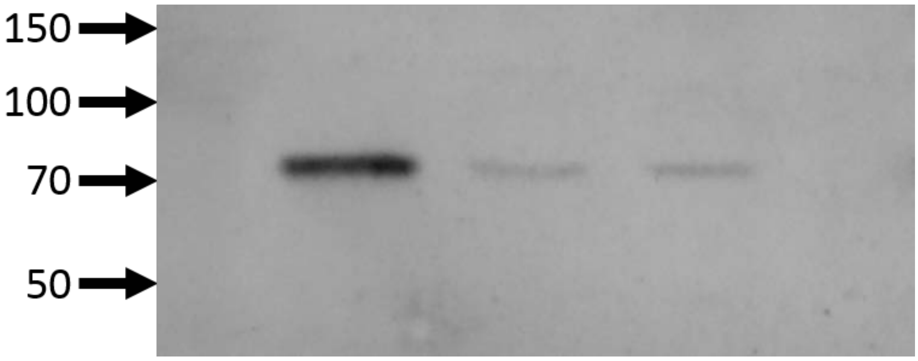

COL23A1 expression in A-498 (siNC, siRNA#1 and siRNA#2)

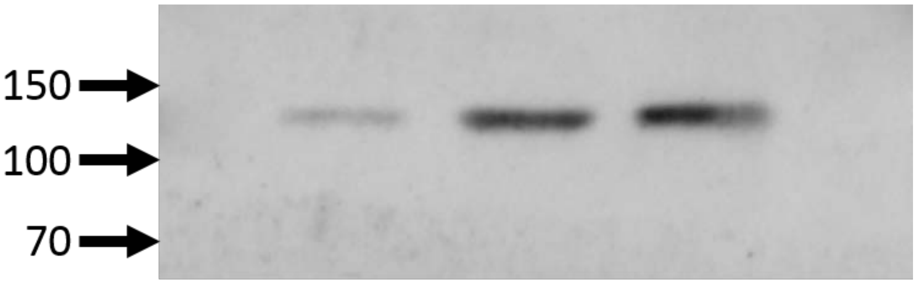

E-cadherin expression in A-498 (siNC, siRNA#1 and siRNA#2)

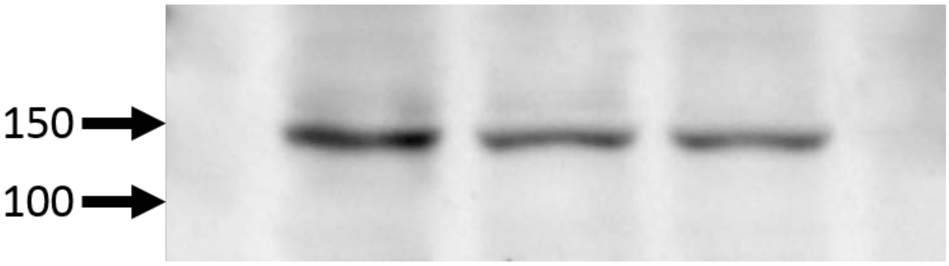

N-cadherin expression in A-498 (siNC, siRNA#1 and siRNA#2)

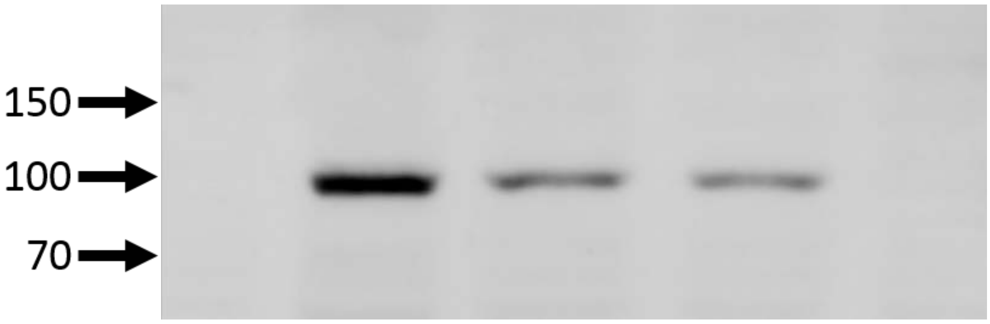

α -catenin expression in A-498 (siNC, siRNA#1 and siRNA#2)

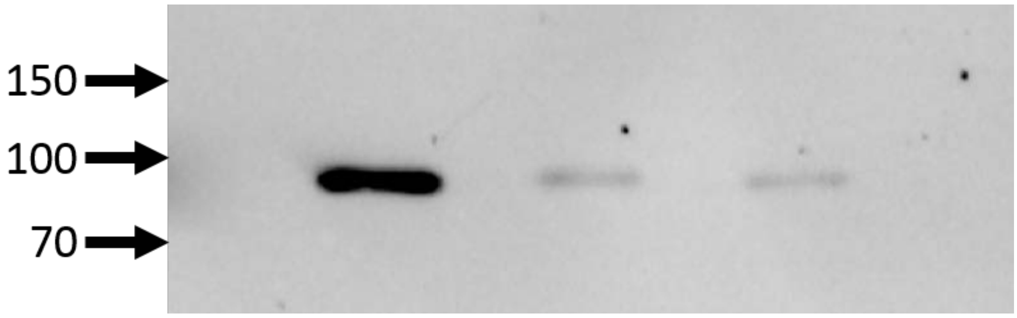

β -catenin expression in A-498 (siNC, siRNA#1 and siRNA#2)

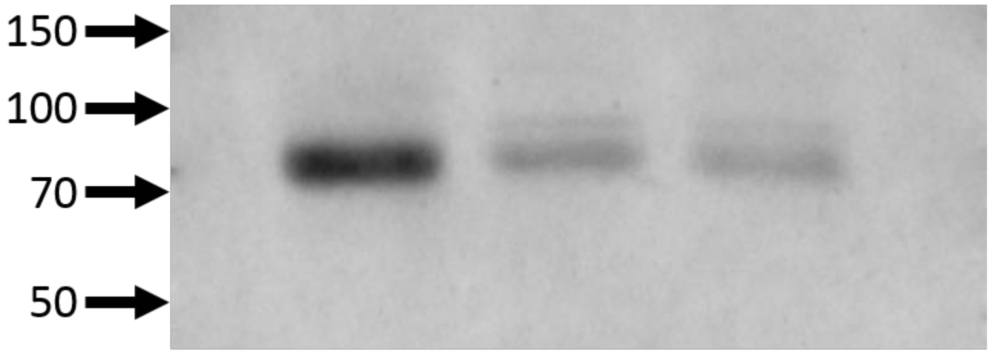

γ -catenin expression in A-498 (siNC, siRNA#1 and siRNA#2)

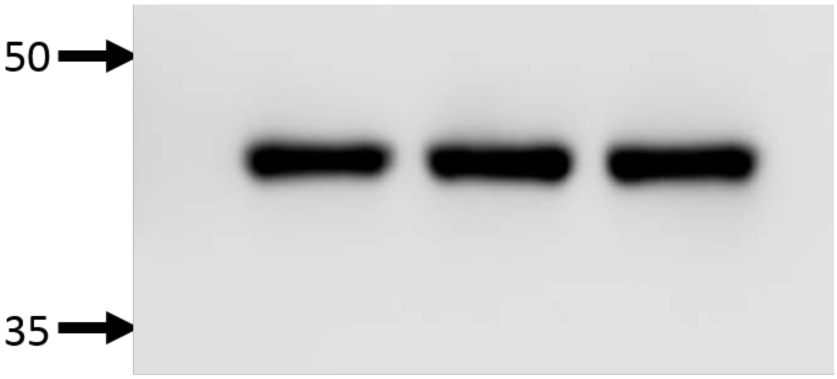

Expression of endogenous control (β-actin) in A-498 (siNC, siRNA#1 and siRNA#2)
